# Affective Congruence between Sound and Meaning of Words Facilitates Semantic Decision

**DOI:** 10.3390/bs8060056

**Published:** 2018-05-31

**Authors:** Arash Aryani, Arthur M. Jacobs

**Affiliations:** 1 Department of Experimental and Neurocognitive Psychology, Freie Universität Berlin, Habelschwerdter Allee 45, D-14195 Berlin, Germany; ajacobs@zedat.fu-berlin.de; 2 Centre for Cognitive Neuroscience Berlin (CCNB), D-14195 Berlin, Germany

**Keywords:** sound-meaning mappings, sound symbolism, effect of sound on meaning, semantic decision task, neurocognitive poetics, processing fluency, poetry

## Abstract

A similarity between the form and meaning of a word (i.e., iconicity) may help language users to more readily access its meaning through direct form-meaning mapping. Previous work has supported this view by providing empirical evidence for this facilitatory effect in sign language, as well as for onomatopoetic words (e.g., cuckoo) and ideophones (e.g., zigzag). Thus, it remains largely unknown whether the beneficial role of iconicity in making semantic decisions can be considered a general feature in spoken language applying also to “ordinary” words in the lexicon. By capitalizing on the affective domain, and in particular arousal, we organized words in two distinctive groups of iconic vs. non-iconic based on the congruence vs. incongruence of their lexical (meaning) and sublexical (sound) arousal. In a two-alternative forced choice task, we asked participants to evaluate the arousal of printed words that were lexically either high or low arousing. In line with our hypothesis, iconic words were evaluated more quickly and more accurately than their non-iconic counterparts. These results indicate a processing advantage for iconic words, suggesting that language users are sensitive to sound-meaning mappings even when words are presented visually and read silently.

## 1. Introduction 

Classic linguistic approaches to meaning embed a core assumption that the way a word sounds does not play any contributing role in its meaning [1]. Rather, language users would access the meaning of words solely through learned, and per se, arbitrary links between linguistic symbols and their cognitive representations. Recent findings, however, support a more differentiated view by acknowledging the importance of non-arbitrary sound-meaning mappings in language processing and in the organization of vocabulary (see [2,3,4] for reviews). These findings distinguish between two types of motivations for such sound-meaning mappings [3]: iconicity, which is based on similarities between aspects of sound and aspects of meaning (e.g., onomatopoeia), versus systematicity, which is based on statistical regularities in language that link specific patterns of sound to specific semantic or grammatical concepts [5,6,7]. However, in many cases, the nature of the relationship between sound and meaning is not particularly clear. The phonaestheme /sn-/ appearing as an initial sound cluster in many English words related to “mouth” or “nose” may serve to illustrate this issue. It is an ongoing debate whether in this case a specific (nasal) quality of the sound of /sn-/links this sound to the concepts of “mouth” or “nose”, or if rather the organization of the vocabulary has evolved in a way so that this specific sound cluster over-proportionally appears in words that are related to these concepts.

In the present study, we aimed at investigating iconicity and its potential facilitatory role in lexico-semantic processing. In addition to a direct acoustic mapping, as in the case of onomatopoeia, iconic words can also evoke other sensory (including visual and tactile), motor, or affective experiences by systematically relating properties of such experiences to phonetic features or acoustic properties [4,8] as evident in ideophones (e.g., “twinkle”, [9,10,11]), in mimetic words [12,13], or in affective responses associated with the phonology of swear words [14,15]. Owing to such a sound-meaning mapping, iconic words have been suggested to be capable of directly evoking sensory, motor, or affective experiences by systematically relating properties of such experiences to phonetic features or acoustic properties of words [4,8,15,16]. 

From a learning perspective, empirical evidence for both children and adults support an iconic advantage for learning the vocabulary of a language with which they had no prior experience. For instance, the meaning of Japanese iconic verbs, compared to non-iconic verbs, have been shown to be better learned and generalized by English speaking children [17,18,19]. These results are in line with the analyses of longitudinal diary data which suggest that over the course of language development iconic words are in general acquired earlier and potentially employed by infants as a bootstrapping mechanism on both lexical and phonological levels [20,21,22].

By the same token, as in vocabulary learning, iconicity has shown to facilitate language processing. Particularly, in sign languages, in which iconic relationships between form and meaning are far more prevalent than in spoken languages [23,24], iconicity has been shown to facilitate a variety of language processing tasks such as picture–sign matching, phonological decision, and picture naming [16], indicating that during lexical processing, iconic words benefit from an additional path between form and meaning by activating conceptual features related to perception and action (see also, [22]). Also, onomatopoetic words imitating animal sounds (e.g., “cuckoo”) have been shown to recruit brain regions involved in the processing of both verbal and nonverbal sounds [25]. These findings indicate that iconic words profit from additional processing networks that can facilitate both vocabulary learning and lexical processing [3,18].

Nevertheless, unlike pioneering works on the facilitatory effect of iconicity in sign language [16,22] which also laid the groundwork for the theoretical framework of such investigation, related research on spoken language still faces some limitations. Previous work on the processing advantage of iconicity in lexico-semantic processing of spoken language has so far mainly focused on either nonwords [26,27], onomatopoeia, and ideophones, including Japanese mimetic words [9,10,11,28], or on cases typically considered as systemticity [6,7,29]. Therefore, empirical evidence on whether iconic mappings in a real word can in general facilitate lexico-semantic processing is missing. This is chiefly due to a lack of appropriate measures for both the sound and meaning aspects of words. This limitation has prevented previous research on real spoken words to move beyond onomatopoeia and ideophones, leaving open the question of whether iconicity could be considered a “general” mechanism facilitating language processing. In addition, due to the limited number and the specific properties of onomatopoetic words and ideophones (e.g., phonological construct, frequency, etc.), no empirical research has so far investigated the effect of iconicity on lexico-semantic processing in a carefully controlled experimental paradigm. In the present investigation, we aimed at extending the results of previous works to the facilitatory effect of iconicity in “ordinary” words during a semantic decision task. 

By capitalizing on the affective domain, in a recent study, Aryani et al. [15] provided quantitative measures for *lexical* affective meaning and *sublexical* affective sound of words in a two dimensional space of valence (ranging from pleasant to unpleasant) and arousal (ranging from calm to excited), with both measures empirically validated at behavioral and neurobiological levels of analysis (see [30] for the lexical and [15,31] for the sublexical measure). The results of the large-scale lexicon analysis suggest that affectivity in the implicit sound of printed words can influence the listener in their judgment about the words’ affective meaning. In the present study, we aimed at extending the scope of the above mentioned work and categorized word in two groups of iconic vs. non-iconic based on the congruence between sound and meaning in the affective domain. We asked whether iconicity can facilitate evaluative decisions on words’ affective content: Imagine two words representing similar lexical affective content (e.g., both high arousing), but one sounds harsh (congruent with the meaning) while the other sounds soft and calming (incongruent with the meaning). Which one will be classified more quickly and more accurately as high arousing in a decision task on affective meaning? A null hypothesis (H0), according to the established notion of linguistic arbitrariness [1], will expect no significant differences, while our alternative hypothesis (H1) predicts iconic (i.e., congruent) words to be evaluated more quickly and more accurately than non-iconic (i.e., incongruent) words [4,8,16]. This prediction is supported by previous findings on multimodal emotional convergence that suggest presentation of congruent bimodal emotional cues (e.g., verbal and nonverbal) yield faster and more accurate emotion judgments than unimodal presentations (e.g., only verbal) [32,33,34].

**Figure 1:**
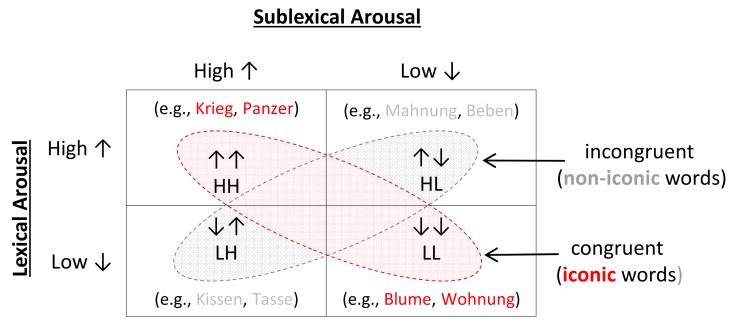
Words were organized in a 2 × 2 design with each of experimental factors (*lexical arousal* and *sublexical arousal*) manipulated in two distinct groups consisting of extreme levels of arousal (High = exciting, and Low = calming). The congruence vs. incongruence of *lexical arousal* (meaning) and *sublexical arousal* (sound) resulted in two groups of iconic vs. non-iconic words, respectively. Two example words (in German) from each category are given in each cell.

To test this hypothesis, we focused on the affective dimension of arousal and organized words in two groups of iconic and non-iconic by the orthogonal manipulation of the factors *lexical arousal* and *sublexical arousal* (Figure 1). In a two-alternative (high arousing vs. low arousing) forced choice task, we then asked participants to decide as quickly and accurately as possible whether the meaning of visually presented words was “exciting” or “calming” (i.e., an arousal decision task). Note that at both *lexical* and *sublexial* levels our experimental design involves primarily the manipulation of arousal rather than valence. At the *sublexical* level, arousal plays a dominant role in models of vocal emotion communication [35,36] and in shaping affectivity in a word’s sound [15]. At the *lexical* level, the first emotional appraisal of a stimulus has shown to be related to arousal which qualifies it as the primary factor producing emotional interference in information processing tasks [37,38,39]. Thus, with regard to rather faster sensory processing of words’ sound, arousal seems to be a more suitable candidate for an interactive effect between sound and meaning. Note that since the decision response time for a forced choice task had to be measured accurately, words in this study were presented visually. Therefore, it is important to mention that the use of the term “sound” in the present work refers to the implicit sound of words derived from phonological and prosodic recoding [40,41,42].

## 2. Materials and Methods

The study was approved by the ethics committee of the Freie Universität Berlin and was conducted in compliance with the Code of Ethics of the World Medical Association (Declaration of Helsinki). All participants gave their consent in written form prior to participating in the study.

### 2.1. Stimuli

One hundred and sixty nouns (one to three syllables long) were selected for a 2 × 2 design involving twofold manipulations of *lexical* and *sublexical arousal* (see Figure 1). For *lexical arousal*, we used ratings for words’ affective meaning (min = 1: very low arousing, max = 5 very high arousing) from the normative database BAWL-R [43]. *Sublexical arousal* was calculated using the recent psychoacoustic model [15]. This model is based on specific extracted acoustic features of pseudowords (e.g., pitch, formants, and intensity) that predict ratings given on the affectivity of their sound (see study2b in [15]). 

Words were then divided into two distinctive conditions of “high” and “low” arousing for each of factors: *lexical arousal* (“high” > 3.25, “low < 2.75) and *sublexical arousal* (“high” > 3, “low” < 3) and carefully matched for relevant psycholinguistic factors (see Table 1).

Due to a natural confound between affective arousal and valence, words in the condition of “high” *lexical arousal* were more negative in valence than words in the condition “low” *lexical arousal*. In order to prevent participants to build an alternative strategy basing their decision on valence rather than arousal, 60 filler words with the rather rare combination of high *lexical arousal* and positive *lexical valence*, as well as 60 words with low *lexical arousal* and negative *lexical valence* were added to the stimulus set, which were excluded from further analyses.

### 2.2. Participants

Thirty-six right-handed German native speakers (26 women, mean age: 22.5 years, range: 18–34 years) with no history of neurological or psychiatric illness volunteered to participate in the study, receiving either five Euros or psychology course credit for their participation. All participants reported normal or corrected-to-normal vision and provided written informed consent to participate in the study. Handedness was determined using the Edinburgh Inventory [44]. 

### 2.3. Procedure

Participants were instructed to decide, as quickly and correctly as possible, whether the meaning of a word presented visually was either high or low arousing (exciting or calming), and to correspondingly press one of two designated buttons on the keyboard (in German: “*Deine Aufgabe ist es, so schnell und so korrekt wie möglich zu entscheiden, ob du die Bedeutung des präsentierten Wortes als aufregend oder beruhigend empfindest….Für deine Entscheidung verwende bitte die beiden Tasten (...) für aufregend und (...) für beruhigend*”). The assignment of the response buttons was counterbalanced across participants. Participants worked through 10 practice trials before starting with the 280 (160 experimental + 120 distractors) main trials. Each trial started with a fixation cross in the screen center with a jittered duration between 1.5 and 3 s and continued with the stimulus item being presented for 1.5 s or until a decision was made. The order of item presentation was fully randomized. For each item, we recorded the response of the first button press. 

After the decision task, in a separate study, the same participants were asked to rate the same 160 relevant words for *lexical arousal*. Adapting the instructions used for the original BAWL ratings [43], participants were invited to read the presented item and evaluate how exciting or calming the presented word means. The 5-point affective sound of arousal scale ranged from 1 (*sehr beruhigend*/“very calming”) to 5 (*sehr aufregend*/“very exciting”). We also incorporated the self-assessment manikins (SAM) that were used in the ANEW study [45]. The items were randomly presented to minimize primacy or recency effects. We then used these rating values as a reference for evaluating responses given in the decision task, thereby distinguishing between “wrong” responses and “subjectively different” responses. 

### 2.4. Analysis

Trials without response were excluded from the analyses (2%, N = 110). We then compared the responses of each participant with their own affective judgment given in the rating study. Responses in the decision task that were in accordance with the rating values, but not in alignment with the original ratings used in experimental manipulation, or vice versa, i.e., *subjectively different* responses, were excluded from the analyses (17%, N = 1002), leaving 447 *wrong* responses (7%) and 4201 *correct* responses (73%). Using language stimuli, we chose Linear Mixed Model (LMM) analysis—over the classic F_1_-F_2_ test—which provides a solution for the long-standing problem of how to analyze experimental data that contain two crossed random effects, i.e., items and participants (see for instance [46] for a review). RT and accuracy data for the items were analyzed with a linear mixed fixed and random effects model using the statistical software JMP 13Pro (SAS Institute Inc.), with *lexical* and *sublexical arousal* and their interaction as fixed effects and participants and items as random effects. 

In order to ensure that the exclusion of a large amount of responses (none and *subjectively different*) was randomly distributed across experimental conditions and did not bias the results, we took the 1112 excluded words and ran the same mixed model analysis predicting the RT within these excluded items.

## 3. Results

A comparison between original ratings for *lexical arousal* (from the BAWL) and the average of post hoc ratings revealed a high consistence between values: r = 0.94, *p* < 0.0001, indicating the reliability of the used measure for *lexical arousal* as experimental factor. 

The analysis of the excluded responses showed that the distribution of these items across experimental conditions was very similar over congruent (9.8%, N = 568) vs. incongruent conditions (9.4%, N = 544) and not significantly different over participants (*p* = 0.96). Within the excluded items, there was no significant effect of any of the experimental factors on the reaction time nor a significant interaction (all ps > 0.3), suggesting that the exclusion of items did not follow a systematic pattern, and consequently, did not bias the results of the remaining responses.

**Figure 2:**
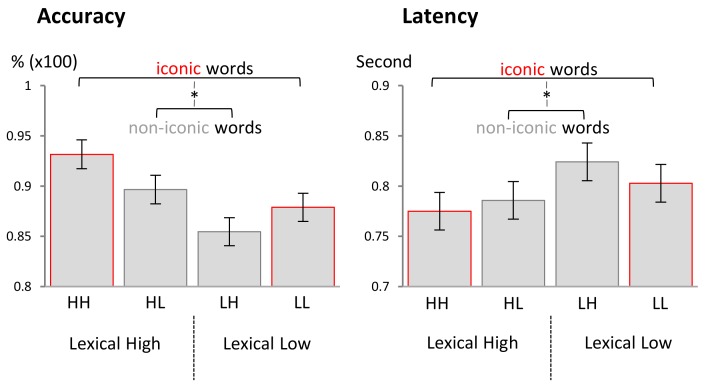
Congruent words (iconic) were classified more quickly (**right**) and more accurately (**left**) in the corresponding lexical group compared to incongruent words (non-iconic).

Results of two main LMM analyses on remaining responses are displayed in Figure 2 and Table 2. A significant effect of *lexical arousal* on accuracy and on RT was observed with lexically high-arousing words classified more correctly and more quickly than low-arousing words (both ps < 0.001). No direct effect of *sublexical arousal* on response accuracy or on RT was observed (*p* = 0.57, *p* = 0.48, respectively). Importantly, there was a significant interaction between *lexical* and *sublexical arousal* for both accuracy and RT (both ps < 0.05). Post hoc analysis showed that within each *lexical* category, iconic words were associated with a higher response accuracy and a shorter RT than non-iconic words (see Table 2 for further results).

## 4. Discussion

In this study, we investigated the effect of iconicity on affective semantic decisions and tested whether language users take the sound aspect implicitly into consideration. In line with our H1, faster latencies and higher accuracy in responses were observed for iconic words, i.e., words that exhibit similarity between meaning and sound in affective domain. Our finding, thus, clearly shows that in the context of language processing, human subjects are sensitive to affective cues that are provided by words’ sound even when they are presented visually and read silently. Such affective cues can be integrated in higher cognitive processes and affect semantic decisions, thereby facilitating the evaluation of words’ affective content when sound and meaning aspects are congruent. Crucially, this effect is evident even when the attentional focus is not directly on the sound aspect of words, suggesting an implicit effect of sound on the evaluation of words’ meaning (see also, [15,47]). With this study, we aimed to build upon the previous results on the facilitatory effect of iconicity in lexico-semantic processing, which has been reported in sign language [16,22], in onomatopoetic words and in ideophones [9,10,11,13]. By using quantitative measures for both sound and meaning of words, we extended the results of previous findings to a larger number of “ordinary” words in the lexicon and in the context of affective meaning.

Also, the important role of multimodal convergence of emotions in making appropriate and faster decisions in emotional evaluation is supported by our data. A major benefit of multimodal integration has been shown to optimize efficient information processing by minimizing the uncertainty of ambiguous stimuli (see [32,48] for recent reviews). This is well in line with our behavioral results, in which words possessing congruent affective information from two different sources (i.e., sound and meaning) were categorized more quickly and more accurately.

The observed effect of lexical arousal on latency and accuracy also supports the previous findings on preferential processing of high arousing compared to low arousing words in decision tasks (e.g., [49]), which is proposed to be rooted in a biologically adaptive response leading to a faster and more accurate evaluation of emotionally relevant stimuli.

Importantly, in line with the results of previous investigations [15,26], the effect of iconicity facilitates lexico-semantic processing of words even when they are visually presented and silently read (see [50] for an ERP study for the effect of implicit sound). Note that visual word recognition generally involves the activation of phonological codes [40,41,42] and language users appear implicitly influenced by affective sound of visually presented words when evaluating the affective meaning of these words [15]. However, as we did not control our stimuli for orthographic features, a possible effect of graphemes on the processing of the affective content of words [51,52] is not precluded.

With the present study, we also aimed at drawing attention to the role of emotion in language processing, and in particular, in the study of iconicity. Focusing only on perceptuomotor analogies between sound and meaning, previous studies often overlooked investigating emotion as a modality of experience similar to sensory and motor processing [15,53,54]. Affective meaning is, however, a fundamental aspect of human communication that have been proposed as the original impetus for language evolution [55,56]. Therefore, from a phylogenetic perspective, the effect of iconicity may be most evident in the affective communication. Here, iconicity serves as an interface for accomplishing the need to map linguistic form onto human affective experience as a vital part of meaning making. When analysing the results, we had to exclude a relatively large number of items (17%) that were differently rated from the original ratings used in the experimental manipulation (i.e., the BAWL ratings). This may call for cautious interpretation of the results as it raises a question about the nature of arousal as a semantic feature. A more detailed analysis of these items did not reveal any specific pattern in regard to the degree of arousal nor to a specific group of words. Previous rating studies have repeatedly shown that ratings of valence are relatively consistent across participants while arousal is much more variable [43,57,58]. It has been suggested that valence is a semantic super-feature that results from an integration of both experiential and distributional data [54] as assumed by the semantics theory of Andrews et al. [59]. Arousal, however, may be derived by way of experience with the physical world and thus being less distributional (i.e., language based) and more experiential (i.e., non-language based). This, in turn, can explain the individual differences of arousal ratings at the level of meaning and, at the same time, its consistence at the level of sound leading to its dominant role in models of vocal emotion communication [35,36] as outlined in the introduction.

Concerning the nature of sound-meaning mapping, two different types of mapping, i.e., iconicity and systematicity, have been suggested in the previous work [3]. The sound-meaning mapping in a word is considered iconic when both sound and meaning independently refer to a similar specific (sensory, motor, or affective) domain [4]. For instance, some swear words are considered iconic because both their sound and their meaning possess negative valence [14]. In the present study, we used two different measures for assessing the sound and meaning of words based on their affective arousal. At the meaning level, our measure for the lexical arousal has been cross-validated in various empirical studies regarding experiential, behavioral, and neurobiological levels of analysis [30]. Also, at the sound level, the measure of sublexical arousal used in this study has been shown to have an inherent affective quality based on acoustic features that are known to modulate nonverbal emotional communication [15] and can evoke affective brain responses similar to other types of affective sounds [31]. Consequently, it is reasonable to conclude that our finding on the facilitatory effect of sound-meaning mapping is related to iconic mappings of words rather than statistical regularities in the lexicon.

Our finding can also help to gain a better understanding of affective and aesthetic processes of literary reading [60,61]. Poetry, for instance, seems to be one of the most promising forms of literature for sound-meaning investigations. The relation of “form” to “feeling” supposedly lies at the basis of poetry [62], and the “*differentia specifica*” of poetry is located in its formal characteristics and iconic properties [63]. Poetry is on the one hand inherently concerned with emotional expressions, and on the other hand, is accompanied by the artful deployment of sound patterns [61,64,65,66,67,68]. In this context, our results on the facilitated lexical processing of iconic words can be linked to previous findings on the notion of processing fluency stating higher ease of processing leads to a higher aesthetic pleasure [69,70]. This may provide additional explanation for the preferential use and the aesthetic effects of stylistic devices such as phonaesthetics and iconicity in poetry. 

## Figures and Tables

**Table 1:** Characteristics of word stimuli.

Variable	Word Category								Inferential Statistics
	HH		HL		LH		LL		
	M	SD	M	SD	M	SD	M	SD	
*Lexical Arousal*	3.86	0.43	3.75	0.35	2.13	0.28	2.17	0.32	F(3,156) = 301, *p* < 0.0001
*Lexical Valence*	−1.59	0.66	−1.44	0.63	1.03	0.67	1.04	0.74	F(3,156) = 190, *p* < 0.0001
*Sublexical Arousal*	3.00	0.25	2.17	0.15	2.97	0.22	2.17	0.14	F(3,156) = 230, *p* < 0.0001
Word Frequency	0.97	0.67	0.88	0.75	0.90	0.74	0.93	0.66	F(3,156) = 0.11, *p* = 0.95
Imageability Rating	4.44	1.27	4.31	1.08	4.32	1.43	4.27	1.37	F(3,156) = 0.12, *p* = 0.94
Number of Syllables	2.13	0.56	2.08	0.41	2.15	0.57	2.08	0.47	F(3,156 = 0.21, *p* = 0.88
Number of Letters	6.05	1.22	6.05	1.20	6.08	1.40	6.00	1.30	F(3,156) = 0.02, *p* = 0.99
Number of Phonemes	5.53	1.20	5.33	1.02	5.45	1.22	5.20	1.07	F(3,156) = 0.64, *p* = 0.59
Orth-Neighbors	1.40	1.69	1.08	1.79	1.45	2.00	1.75	2.06	F(3,156) = 0.85, *p* = 0.46
Orth-Neighbors-HF	0.50	0.91	0.43	1.26	0.48	0.93	0.50	0.99	F(3,156) = 0.04, *p* = 0.98
Orth-Neighbors-Sum-F	0.72	1.06	0.49	0.82	0.69	1.05	0.80	0.88	F(3,156) = 0.76, *p* = 0.51
Phon-Neighbors	1.75	2.51	1.98	3.04	1.93	2.58	2.35	3.34	F(3,156) = 0.30, *p* = 0.82
Phon-Neighbors-HF	0.55	0.88	0.63	1.76	0.55	1.08	0.60	1.24	F(3,156) = 0.03, *p* = 0.99
Phon-Neighbors-Sum-F	0.79	1.02	0.69	1.01	0.67	0.92	0.88	0.88	F(3,156) = 0.40, *p* = 0.75

Note: Sum-F = sum of the frequency of neighbors; HF = number of neighbors with higher frequency than the word itself.

**Table 2:** Results of fixed effects, the interaction term, and the intercept of the mixed model analysis.

Term	Response Accuracy				Response Latency			
	Estimate	Std E	*t*	*p*	Estimate	Std E	*t*	*p*
Intercept	0.903	0.010	90.07	<0.0001	0.797	0.017	45.47	<0.0001
*lexical arousal*	0.018	0.005	3.55	0.0005	−0.016	0.003	−4.32	<0.0001
*sublexical arousal*	0.002	0.005	0.56	0.5784	0.002	0.003	0.69	0.4899
lexical*sublexcial	0.013	0.005	2.55	0.0120	−0.008	0.003	−2.11	0.0369
